# Coating doxycycline on titanium-based implants: Two in vivo studies

**DOI:** 10.1016/j.bioactmat.2020.05.007

**Published:** 2020-06-22

**Authors:** Maryam Rahmati, Ståle Petter Lyngstadaas, Janne E. Reseland, Ingrid Andersbakken, Heidi Straume Haugland, Mónica López-Peña, Antonio Gonzalez Cantalapiedra, Fernando Maria Guzon Muñoz, Håvard Jostein Haugen

**Affiliations:** a Department of Biomaterials, Institute of Clinical Dentistry, University of Oslo, 0317, Oslo, Norway; b Universidade de Santiago de Compostela Facultad de Veterinaria, Campus Universitario, s/n, 27002, Lugo, Spain

**Keywords:** Titanium-zirconium, Doxycycline, Antibiotic, Coating, SLActive

## Abstract

Regardless of the substantial progress in designing titanium-based dental implants and aseptic techniques, infection remains as the most common complication after implantation surgeries. Although, having a weakened immune system or systematic diseases is not seen as contraindicated for dental implants anymore, controlling the immune system is required to avoid surgical site infections after implantation. These patients have to control the surgical site infections by taking a high daily dose of oral antibiotics after dental implantation. The antibiotics oral administration has many side effects such as gastrointestinal symptoms, skin rashes and thrush. Coating antibiotics on the biomaterials surface could be a promising solution to reduce these disadvantages through locally releasing antibiotics in a controlled manner. The aim of this study was to investigate the effects of doxycycline coating layer on titanium-zirconium alloy surfaces *in vitro* and in vivo. In our previous studies, we demonstrated the chemical presence of doxycycline layer *in vitro*. In this study, we examined its physical presence using field emission scanning electron microscope and confocal microscope. We also analyzed its controlled released manner using Nano-Drop UV Vis spectrometer. After *in vitro* characterization of the coating layer, we evaluated its effects on the implant osseointegration in dogs and rabbits. The histological and histomorphometrical results exhibited no significant difference between doxycycline coated and uncoated groups regarding the implants osseointegration and biocompatibility for dental applications. Therefore, coating a doxycycline layer on TiZr implants could be favorable for reducing or removing the antibiotics oral administration after the implantation surgery.

## Graphical abstract

**Figure:**
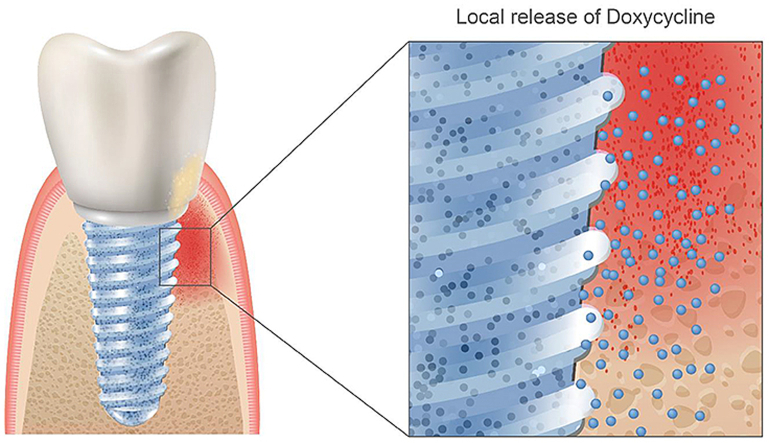

## 1 Introduction

Replacing the missing teeth with titanium implants is an established treatment in dentistry [1]. Titanium and its alloys are the materials of choice in dentistry owing to their high mechanical strength and corrosion resistance [2,3]. Over the past few years, dentists have used titanium-zirconium (TiZr) implants for achieving higher fracture toughness and corrosion resistance compared to titanium implants [[4], [5], [6], [7]]. Although adding zirconium significantly improves the success rate of dental titanium-based implants [8,9], avoiding the acute surgical site infections in some patients is still challenging [[10], [11], [12]]. For instance, a weakened immune system, systematic disorders, tobacco addiction, and head or neck radiotherapy are several risk factors that could lead to dental implants contraindication because of possible wound healing disruptions and/or high risk of infections [13]. Researchers have suggested coating therapeutic agents on the biomaterials surface to locally release antibiotic drugs in a controlled manner [14,15]. Coating a layer of antibiotics on dental implants might also improve osseointegration and reduce the infection rates [15]. After implantation, the adsorption of water, ions, proteins and biomolecules on the implant surface affects subsequent cellular responses such as cell adhesion, proliferation, immigration, and differentiation [16]. As the implant surface chemical properties directly affect the amount and composition of adsorbed biomolecules and proteins on the surface, coating a layer of antibiotic agents (such as doxycycline, gentamycin, cephalothin, amoxicillin) can chemically improve molecular and cellular responses to the surface [17,18].

Among these antibiotics, doxycycline is one of the most common used antibiotics for controlling infection after dental implantation surgeries [19]. Some *in vitro* studies revealed that in addition to antibiotic properties, doxycycline could be a potential factor in treating periodontal diseases [20,21]. Thus, a direct incorporation of this drug in the implant system could be favorable for controlling its release rates in the implanted site. In our previous studies, we designed a system for doxycycline direct incorporation in the implanted area through its electrochemically binding onto the TiZr alloy surfaces [10,17,19]. Our *in vitro* studies on murine osteoblasts indicated that the coating layer could significantly enhance alkaline phosphatase and osteocalcin gene expression levels after two weeks, with lower cytotoxic effects compared to the implants without doxycycline coating [10,19]. Although there is no doubt in antibiotic properties of doxycycline, its effects on TiZr implants osseointegration and biocompatibility have not yet been fully examined in vivo.

**Fig. 1:**
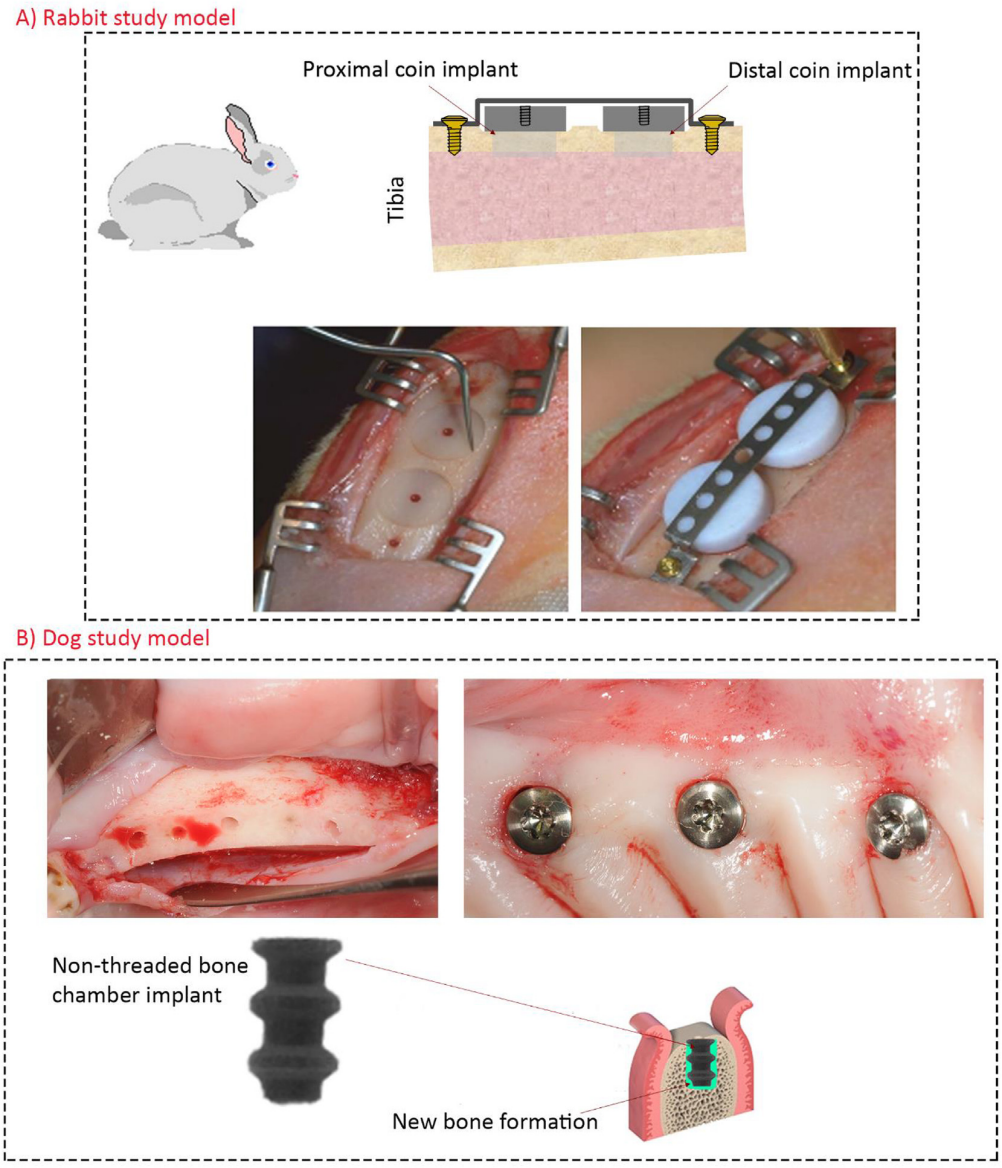
The clinical images of rabbit and dog implantation procedures. **A)** In the rabbit animal model, coin shaped implants were placed onto the cortical bone of tibia without mechanical fixation to the bone. Therefore, the implant was retained inactively on bone by a titanium band retainer. After implantation, the contact between the implants and bone was limited to the flat test surfaces. Additionally, a polytetrafluoroethylene (PTFE) cap was used for covering the vertical and the upper faces of implants to hinder interlocking effects from lateral bone attachments. **B)** Clinical images of dental bone chamber implants in dog animals. Three bone chamber implants were placed per quadrant (six implants per animal jaw). In this study, we used bone chamber implants because the bone formation around this type of implant is only new bone; however, in clinical implants it is a combination of new and old bone formation.

In this study, we aimed to investigate the bone ingrowth and attachment to TiZr implants containing a doxycycline coating layer in both rabbit and dog animal models. The hypothesis of this study was that coating a layer of doxycycline on titanium-based implants would not worsen then host responses to implants in healthy bone conditions. Firstly, we confirmed the physical presence of doxycycline layer and its controlled released manner *in vitro*. Subsequently, we implanted TiZr coins (n = 32) with or without the doxycycline coating layer into the tibia of eight rabbits for 4 and 8 weeks and investigated the coating effects on the bone attachment to coins. We also examined the bone ingrowth and attachment to bone chamber dental implants in six dogs (Fig. 1B). We placed TiZr bone chamber dental implants (n = 36) with or without doxycycline coatings into the dogs mandible. After 4 weeks, we investigated their effects on both ingrowth and attachment to chamber implants, histologically and histomorphometrically.

## 2 Materials and methods

### 2.1 Materials

In the rabbit animal study, coin shaped samples with a diameter of 6.25 mm and a height of 2 mm were used. In the dog animal study, bone chamber implants with a diameter of 3.5 mm and a height of 6 mm were used. All samples were made of Roxolid® TiZr with a zirconium content of 13%–17% zirconium and the surfaces were comparable to the commercially available SLActive® surface (Institut Straumann AG, Basel, Switzerland) [22].

### 2.2 Coating procedure

We briefly explain the coating procedure; however, its details can be found in our previous studies [10,17,19]. Before coating, the test samples (coin and bone chamber implants) were unpacked under laminar flow and washed for 5 min in an ultrasound bath with deionized reverse osmosis water. For the biomolecule-coated samples, 200 mg of doxycycline was dissolved in the buffer to obtain a final concentration of 1 mg/ml. Yangzhou Pharmaceutical delivered doxycycline Hyclate (Yangzhou Pharmaceutical Co Ltd, Jiangsu, China). The authors performed the coating on a custom-made setup, controlled by the software LabView (National Instruments, Austin, TX, USA), providing a constant direct current of 0.65 mA on individual channels for each sample. Thereby, the TiZr samples were connected to the system's cathode, whereas a platinum anode served as the counter electrode. After completing the procedure, all samples were kept under cover gas before analysis, as the exposure to air results in the attraction of environmental carbon, which can impair surface chemistry analysis [23].

### 2.3 Coating characterization

#### 2.3.1 Surface roughness and morphology

In our previous *in vitro* studies we confirmed the chemical binding and composition of the deposited coatings using a PerkinElmer Spectrum 400 fourier-transform infrared spectroscopy (FTIR)/FT-NIR spectrometer (PerkinElmer, Waltham, MA, USA) and an X-ray photoelectron spectroscopy (XPS; Axis UltraDLD XPS spectrometer, Kratos Analytical, Manchester, UK) using monochromatic Al Kα X-rays (hν = 1486.69 eV) [17]. Here, we imaged the implants surface morphology and topography using a Quanta 200 FEG (FEI Hillsboro, Oregon, USA) field emission scanning electron microscope (FE-SEM) and a Leica SP8 upright confocal microscope fitted with HyD and PMT detectors, using oil immersion objective 40x HC PL Apo CS2 40x/1.3 [17].

The surface morphology was measured using a blue light profilometer (Sensofar Pll 2300, Terrassa, Spain) with a 50x objective (50× PI, Nikon, Tokyo, Japan) over an extended topography of 2 × 2 images. Subsequently, the following surface roughness parameters including deviation of the surface, also known as surface roughness (Sa), skewness of the height distribution (Ssk), kurtosis of the height distribution (Sku), core fluid retention index (Sci) were obtained with an advanced software (Sensomap, Sensofar, Terrassa, Spain). Then, the entire surface was examined by FE-SEM for obtaining SEM-images and sputtered all samples with platinum prior to imaging. Its Schottky field emission gun (FEG) allowed high spatial resolution.

#### 2.3.2 Doxycycline release profile

In our previous studies, we used dionized water, phosphate-buffered saline (PBS) and 60% acetonitrile and 3% trifluoroacetic acid (ACN–TFA) to examine the doxycycline release manner from implant surface [10,17,19]. The results from these studies indicated that doxycycline release is very difficult in water, takes several weeks in PBS and takes one week in CAN-TNF [10,17,19]. Because we observed a significant release in CAN-TFA buffer after 6 h and 7 days [10], in this study, we performed another release study in an acetate buffer solution with the same timeline to investigate whether the release manner is the same in different acidic environments or not. A Nano-Drop UV Vis spectrometer (Nano-Drop ND 1000 Spectrometer Technologies, Wilmington, DE, USA) at 263 nm wavelength was used to test the doxycycline release manner. A calibration curve was recorded at doxycycline concentrations between 0.005 mg/ml and 0.25 mg/ml. The drug release was tested in a freshly made acetate buffer solution with pH 3 mixed of acetic acid and sodium acetate (identical to the electro-coating buffer but without doxycycline). All samples were transferred to Eppendorf tubes and added 0.5 ml release buffer. Then, the test was performed in a tabletop shaker (IKA Schüttler MTS 2, VWR, Oslo, Norway) at 300 rpm at room temperature. At predetermined intervals, the solution was removed from the sample and replaced it with fresh release buffer. The buffer samples were stored in Eppendorf tubes in the refrigerator (4 °C) until spectrophotometric analysis started. The scheduled time-points for analyzing the amount of released doxycycline were 1h, 3h, 6h, 24h, 72h, and 7 days.

### 2.4 Animal models and implantation procedures

#### 2.4.1 Rabbit animal model and implantation procedure

We performed the surgery on eight Grey Bastard Chinchillas rabbits (6 months old, weight 2.86 kg, Charles River Laboratories International, Inc, Research Models and Services, Sulzfeld, Germany). The study protocol was approved by the Regional Committee for Research Ethics, Oslo, Norway (2013/1827/REK sør-øst C). The procedures were performed according to the Animal Welfare Act of June 1 2010, No 94 and Regulation on Animal Experimentation of January 15 1996. The animals were kept in cages two days post-surgery, and one day prior to euthanization. The room temperature, humidity and diet were standardized. The sedation and anesthesia were done according to the Norwegian School of Veterinary Science, Laboratory Animal Unit; SOP on anesthesia of rabbits. Rønold et al. [24] described the details of all surgical procedures regarding this animal model for evaluating titanium-based implants [24]. The only difference to this process was the integration of a central defect, drilled into the bone marrow region, with a diameter of 3.5 mm as described by Haugen et al. (Fig. 1A) [25]. In this animal model, one can evaluate the functional attachment of implants *in situ*, with minimal effects of interlocking and shear forces [24].

#### 2.4.2 Dog animal model and implantation procedure

##### 2.4.2.1 Dog animal model

The surgery was performed on 6 Mongrel Hound type female dogs (mean age 15 months, weight 23-27 kg, Marshall Bioresources, North Rose, NY, USA) in Rof Codina Foundation research facility (Cebiovet, Faculty of Veterinary Medicine, Lugo, Spain), according to the Spanish and European Union regulations. The study protocol was approved by the Ethics Committee of the Fundación Rof Codina (AE-LU-001/01/INV.MED.02/OUTROS(04)/15-12). A total of 3 bone chamber implants were placed into predrilled cylindrical cavities of height 6 mm and per quadrant (6 implants per animal jaw). A veterinarian accredited in laboratory animal science monitored dogs daily during the entire study procedure. The experimental segment of the study started after a 3-week adaption period to determine their general health status. The animals were kept in a group kennel with an indoor (15 m^2^) and outdoor (20 m^2^) area, with natural light, air renewing and controlled temperature to 18 ± 2 °C in the indoor area. The animals were fed using a granulated dog diet twice a day with individual bowls and free supply of water.

##### 2.4.2.2 Dog implantation procedure

Mucoperiosteal flaps were reflected bilaterally in mandible. We removed carefully teeth from premolar 1 to molar 1 after root sectioning and accomplished wound closure using mattress sutures for allowing the implanted sites to heal for 4 weeks. Prophylactic administration of cefovecin sodium (8 mg/kg body weight S.C. S.I.D., Convenia®, Zoetis, Spain) were performed postoperatively (single injection provides 14 days of antibiotic half-life). Three implants were prepared bilaterally, using a low-trauma surgical technique under copious irrigation with sterile 0.9% physiological saline (surgery protocol by Institut Straumann AG, Basel, Switzerland). Thereafter, the bone chamber implants were pushed using a custom-made surgical guide. Since the bone chamber are not threaded implants, achieved primary stability was achieved by mechanical retention of the outer implant diameter (Fig. 1B).). During the first two weeks, disinfected using gauzes soaked in a 0.12% chlorhexidine solution (Perio‐Aid Tratamiento®, Dentaid, Barcelona, Spain). The plaque control regimen (3x per week) were performed throughout the study using a toothbrush together with a 0.2% chlorhexidine gel (Chlorhexidine Bioadhesive Gel, Lacer, Barcelona, Spain). The animals were euthanized 4 weeks after bone chamber placement by an overdose of sodium pentobarbital 3%. Thereafter, the mandibles were dissected and fixed in 4% neutral buffered formaldehyde solution for the following histological experiments.

### 2.5 Micro computed tomography analysis

**Fig. 2:**
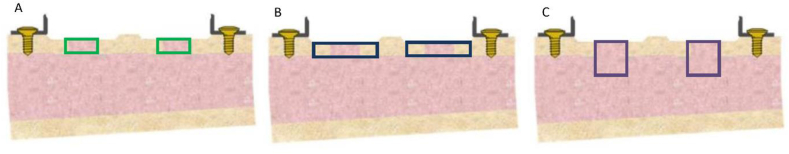
A schematic drawing of the rabbit model with a cortical defect of 3.5 mm diameter. Three different Volume of Interest (VOI) (a, b and c) were evaluated by micro-CT. The green box shows the VOI-1 of 3.5 diameter and 2.5 mm height (**a**), the blue box shows the VOI-2 of 6.2 diameter and 2.5 mm height (**b**) and the purple blue box shows the VOI-3 of 6.2 diameter and 2.5 mm height (**c**).

After sampling the wound fluid following the pull-out test [17], all tibia tissues (n = 32) from the rabbit study were fixated in 4% neutral buffered formaldehyde solution. The coin implants from the rabbit study were scanned using a desktop micro-CT scanner (Skyscan, Aartselaar, Belgium), details of the scanning and image processing can be found elsewhere [10]. In general, the same VOI were chosen for each sample, a cylinder with a diameter of 3.5 mm and a height of 2.5 mm (Fig. 2), starting from the outer cortical bone defect (which was in contact with the implant surface) toward the marrow space. This VOI is identical to the defect created in the cortical bone prior to placement of the samples. The threshold level was set from 65 to 255. In our previous paper, we reported the statistics of bone 3D architectural parameters [10], whereas here we present the 3D cross-sectional images of both coated and uncoated groups after 4 and 8 weeks in the rabbit study.

### 2.6 Histological preparation of tissues

Radiographs were taken in bucco-lingual direction to identify the exact implant position. The tissues were removed and transferred to 4% neutral buffered formaldehyde solutions. After dehydration in a graded series of ethanol (70–100%), the tissues were embedded in Technovit 7200VLC (Kulzer:EXAKT, Kulzer & Co GmbH, Germany). Histological sections were prepared using the Exakt cutting and grinding system, respectively to the cutting and grinding technique described by Donath & Breuner and Roher & Schubert [26]. After sectioning and polishing the slides, Hematoxylin and Eosin (H&E) staining was used for rabbit tibia tissues and LEVAI LACZKÓ staining was used for dog tissues [27].

### 2.7 Histological analysis of tissues

#### 2.7.1 Histological and histomorphometrical analyses of rabbit study

High-resolution images of histological sections were acquired using an automated slide scanner system (Axio Scan Z1, Carl Zeiss Microscopy, Munich, Germany). Images were inspected using the Zen Lite Blue software (Carl Zeiss Microscopy). Before taking photos, both the proximal and distal end sides were defined for each slide. Photos were taken by focusing on the defect area with an overlapping technique of the two-implant sites. The overlapping images were examined using 3 different parameters: Horizontal dimension (index A) of regenerated peri-implant cortical bone, vertical dimension (index C) of new peri-implant cortical bone, and woven (w)/non-woven bone (nw) [28].

Index A: the horizontal dimension of regenerated bone.

A1: No osseous bridge.
A2: Regenerated osseous bridge <50% of the distance between the boarders of defect.
A3: Regenerated osseous bridge >50%, but <75% of the distance between boarders of defect.
A4: Regenerated osseous bridge 75-100% of the distance between the boarders of defect.

Index C: vertical dimension of new peri-implant cortical bone.

C1: <25% of the vertical height of the bone marrow compartment and vast majority of the voids between the filled granules with bone.
C2: 25-75% of the vertical height of cortical bone of the cortical part of the defect regenerated.
C3: >75% of the vertical height of the cortical part of regenerated defect.

w = woven bone: randomly organized collagen fibers.

nw = non-woven bone: collagen fibers organized in parallel layers (lamellar bone).

#### 2.7.2 Histological and histomorphometrical analyses of dog study samples

We stained the 50 μm prepared sections with Levai Laczko for both histology and histomorphomety analyses. A motorized stage transmission light microscope and a PC-based image capture system were used (BX51, DP71, Olympus Corporation, Japan) for analyzing the sections. The quantitative histology was performed by a masked examiner using PC-based image analysis programs: Cellsens 1.13 (Olympus Corporation, Japan) and Image Pro-Premier 9.0 (Media cybernetics, Bethesda, MD, USA). We evaluated 2 quantitative parameters:

• Percentage of bone-to-implant contact (BIC%).

BIC% was defined as the percentage of the bone chamber implant surface in direct contact with bone without intervening fibrous layers; • Percentage of the peri-implant bone area (BA%).

We analyzed the relative BA around the bone chamber implant in a rectangular region of interest (ROI).

### 2.8 Statistical analysis

Plot box was used to analyze the effect of doxycycline coating on TiZr implants functions in both rabbit and dog animal models based on the described parameters. The differences between groups was assessed by a non-parametric analysis (One-Sample Signed Rank Test, Mann- Whitney Rank Sum Test, Normality Test (Shapiro-Wilk), Kruskal-Wallis One Way Analysis of Variance on Ranks (ANOVA). SigmaPlot version 13.0 was used and considered the results statistically significant at P < 0.05 level.

## 3 Results

### 3.1 Coating characterization

#### 3.1.1 Surface roughness and morphology

**Fig. 3:**
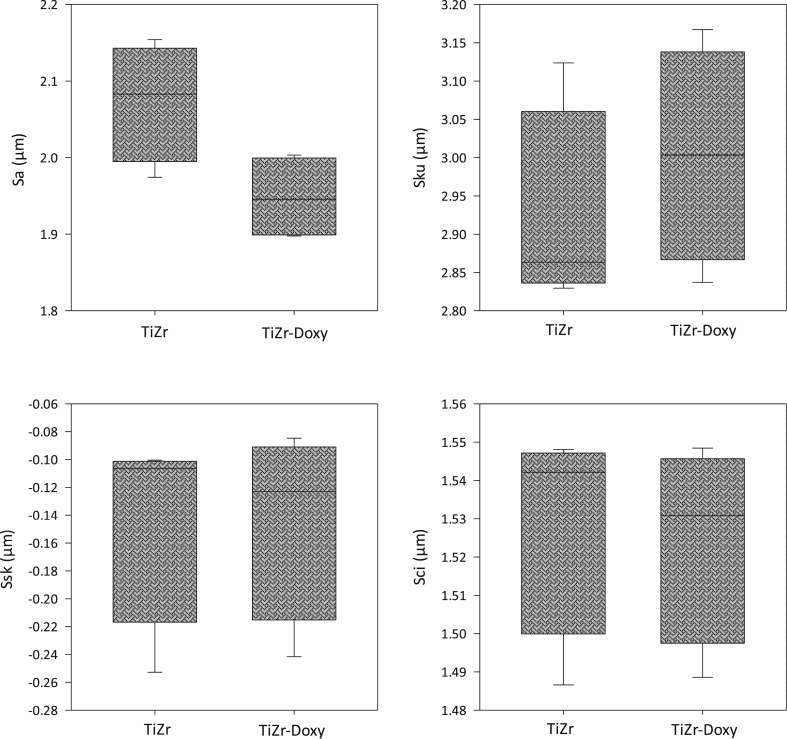
Surface roughness parameters including surface roughness (Sa), skewness of the height distribution (Ssk), kurtosis of the height distribution (Sku), core fluid retention index (Sci) for both doxycycline coated and uncoated TiZr groups. Except Sa parameter, there was no significant difference between doxycycline coated and uncoated TiZr implants for other parameters.

The coating procedure gave a surface that generally was smoother when compared to the uncoated group. Fig. 3 demonstrates the profilometry results of surface roughness parameters including Sa, Ssk, Sku and Sci for both doxycycline coated and uncoated TiZr groups. The one-way ANOVA study revealed that Sa parameter was significantly higher in the uncoated group. Regarding other surface parameters, there was no significant difference between the two examined groups.

**Fig. 4:**
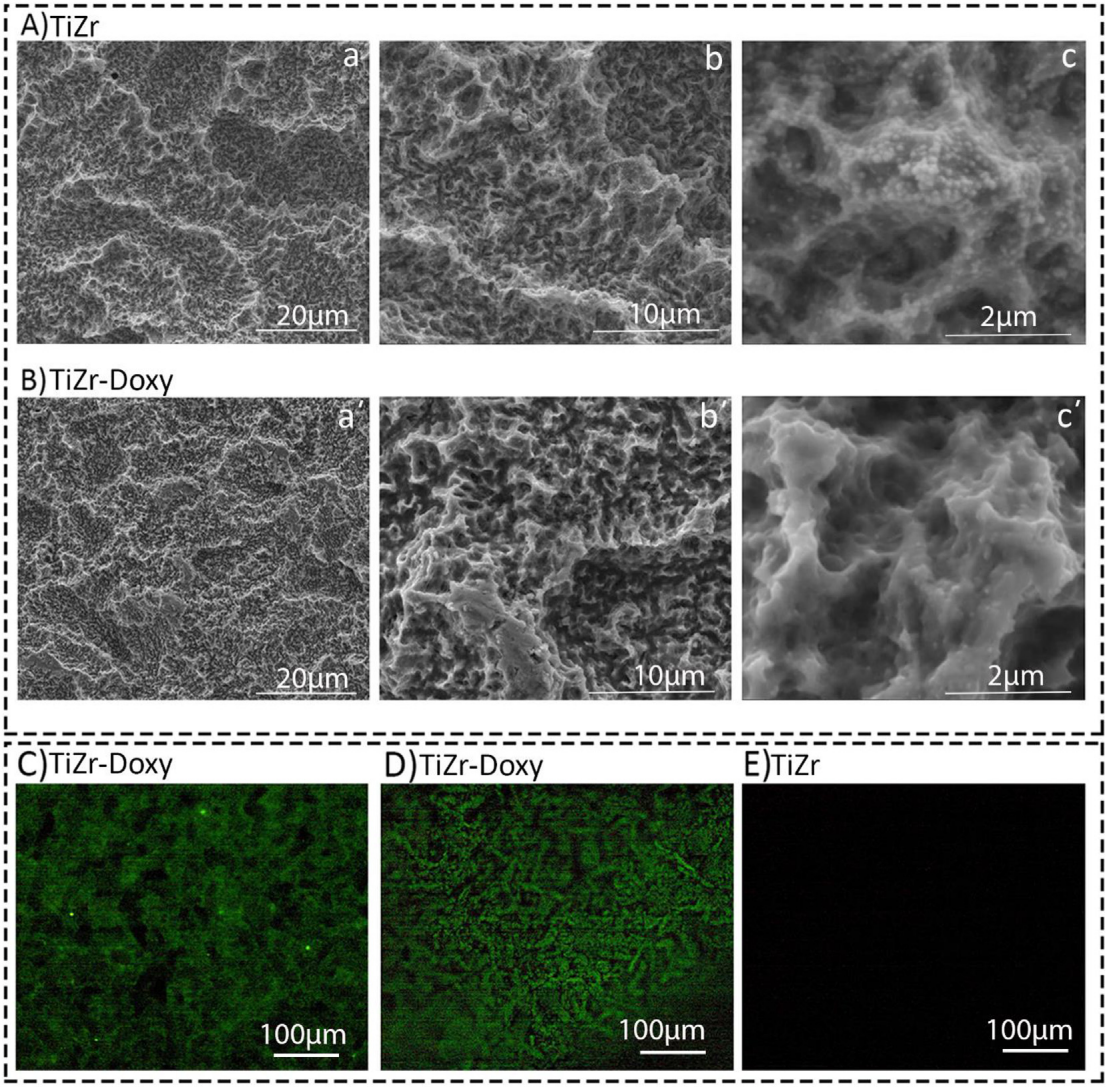
Representative FE-SEM and confocal images for both doxycycline coated and uncoated groups. **A&B)** FE-SEM images for uncoated **(A)** and doxycycline coated **(B)** TiZr implants in three different magnifications including 2000x, 10000x and 50000x. The doxycycline coated TiZr implants had a smoother surface with less sharp edges and peaks compared to the uncoated TiZr implants. **C-D)** Confocal images of both doxycycline coated TiZr coins **(C)** and screws **(D)** confirmed the physical presence of doxycycline layer (green colour) in comparison with uncoated implants **(E)**.

Additionally, Fig. 4A&B shows the FE-SEM images for both uncoated and doxycycline coated TiZr coin implants in three different magnifications including 2000x, 10000x and 50000x. Samples coated with doxycycline showed a generally smoother surface than the uncoated group. Edges and peaks were less sharp. The surface showed very few spherical spots on the surface. In overall, the macrotopography was similar to coated and uncoated surfaces. We also confirmed the physical presence of doxycycline layer on both TiZr coins (Fig. 4C) and screws (Fig. 4D) using a Leica SP8 upright confocal microscope fitted. Compared to uncoated implants (Fig. 4E), we detected a green layer representing doxycycline on the coated TiZr coins and screws surfaces.

#### 3.1.2 2. doxycycline release profile

**Fig. 5:**
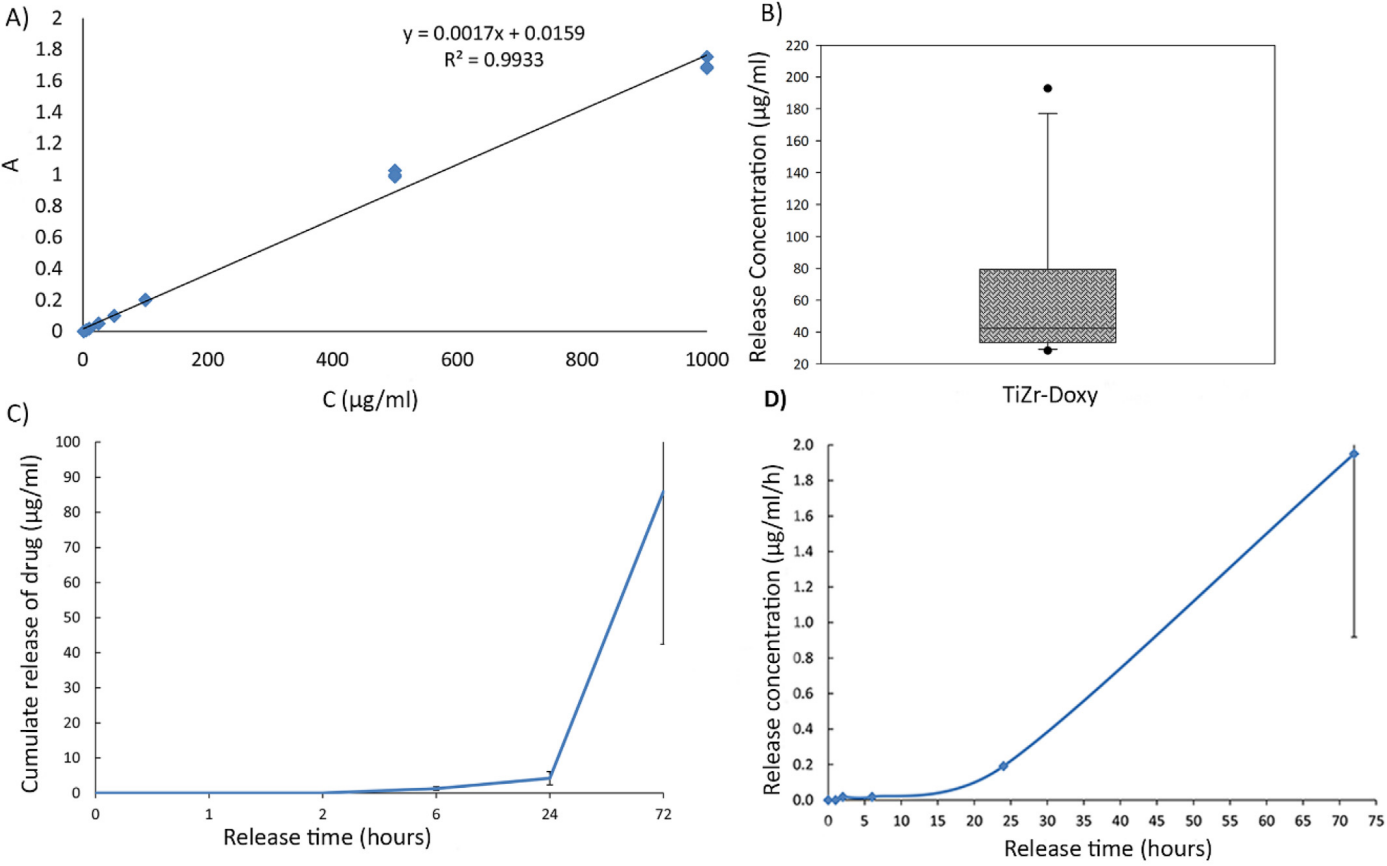
Doxycycline release tests. A) Calibration curve for doxycycline at 224 nm (corrected for absorbance of the sodium acetate buffer) with fitting of R^2^ = 0.8673. B) Mean concentrations of doxycycline after releasing for 72 h at absorbance 224 nm (*p < 0.05, sample numbers = 15). **C)** Cumulative doxycycline release in the release solution at different release time points measured by absorbance at 224 nm with SD. **D)** Doxycycline Release the in release solution per hour measured by absorbance at 224 nm with SD (n = 3). The samples showed a significant release only after 24h indicating that doxycycline is chemically bound to the implant surface.

We used three randomly chosen implants from eight different process batches to measure the drug release profile in five time-points (1h, 3h, 6h, 24h, 72h). The background spectrum was measured (acetic acid/sodium acetate buffer) and subtracted from the data. Different concentrations of doxycycline were used to calculate the calibration curve, with a two-character peak at 350 and 265 nm. In the acetate release buffer, the spectrum shifted toward the UV end of the spectrum with the 265 nm peak shifted to 224 nm, which could be because of doxycycline protonation in the acidic solution. This effect was identical for both coated and uncoated samples. Only negligible amounts of doxycycline released from coated samples during the first 24 h of the study (time-points 1h, 3h, 6h and 24h). However, we observed a significant doxycycline release from samples at 224 nm after 72 h (Fig. 5A–C). Fig. 5D shows that the samples had a mean release of 42 μg doxycycline per mL after 72 h in solution. The delayed drug release from samples indicated that doxycycline was bound or trapped chemically in the hydride layer of the TiZr surface. Under the used acidic conditions in this study, this hydride layer was quite stable without any burst effects. It is reasonable to expect that the release under physiological conditions could be more effective. Because the molecule was stable and retained bioactivity at low pH, the observed protonation of doxycycline was probably reversible and not foreseen as a problem.

### 3.2 Micro computed tomography analysis

**Fig. 6:**
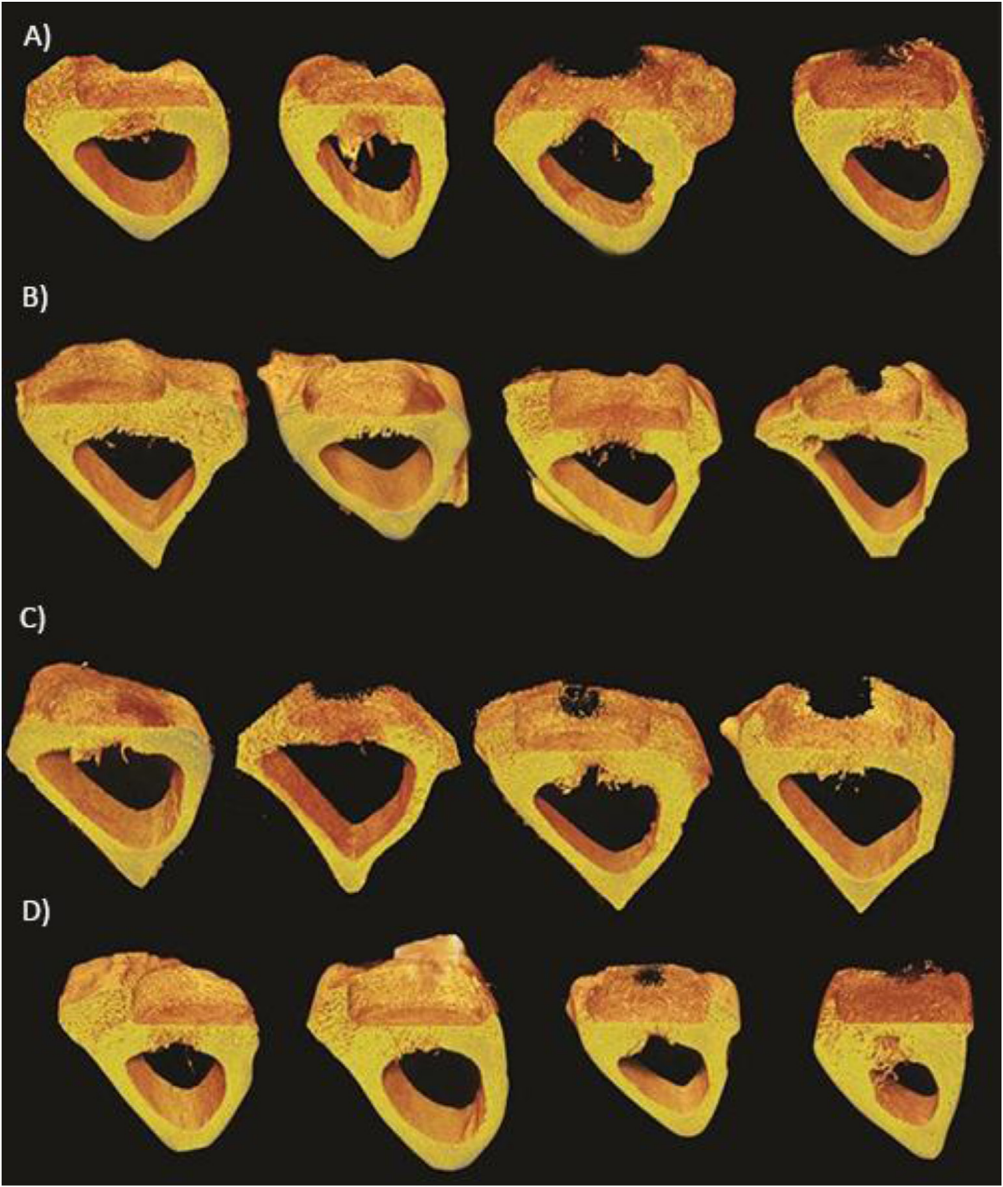
Representative cross sectional 3D micro-CT images in the rabbit study. **A)** Uncoated TiZr implants after 4 weeks healing. **B)** Uncoated TiZr implants after 8 weeks healing. **C)** Doxycycline coated TiZr implants after 4 weeks healing. **D)** Doxycycline coated TiZr implants after 8 weeks healing. Regarding the defect healing, there was no significant difference between uncoated and coated implants.

The cross-sectional 3D images of tibia tissues 4 and 8 weeks after the Implantation surgery exhibited that most defects started to heal in both groups (Fig. 6). The images demonstrated the callus formation up along the coin side in most defects. Compared to the first time point, we also observed more bone for the 8 weeks healing group.

### 3.3 Histological and histomorphometrical analyses of rabbit study

**Fig. 7:**
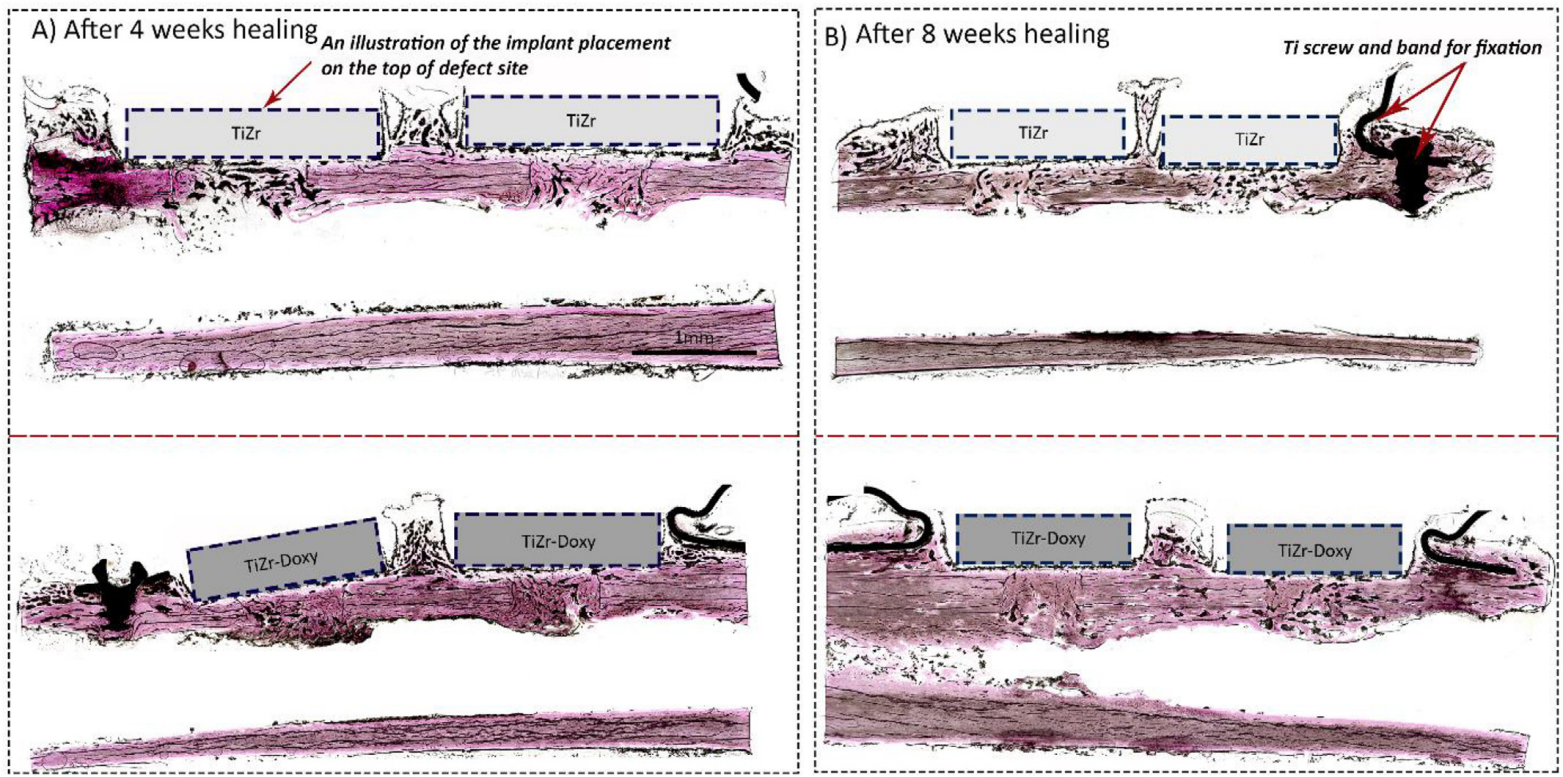
The representative images of H&E staining and histomorphometry results in the rabbit study. H&E staining of doxycycline coated and uncoated TiZr implants 4 **(A)** and 8 **(B)** weeks after implantation in 10x magnification. The rectangles are drawn as an illustration to show the placement of the implants on the top of the defect site during the implantation surgery.

We could not detect any significant difference between doxycycline coated and uncoated groups in both time points regarding their biocompatibility. Fig. 7 shows the representative images of H&E stained tibia rabbits after 4 and 8 weeks healing in both uncoated and coated groups.

The normality test (Shapiro-Wilk) failed for both indexes (P < 0.05). When the normality test failed, we used a “one-way analysis of variance” (ANOVA)-test called “Kruskal-Wallis One Way Analysis of Variance on Ranks”. The test compared two or more independent samples that could have different sample sizes. The differences in the median values among the treatment groups were not great enough to exclude the possibility that the difference was due to the random sampling variability. Regarding the tissue texture, although few TiZr implanted tissues had a non-woven texture after 4 weeks, the majority of tissues in both doxyxycline coated and uncoated groups had a woven texture after 4 and 8 weeks of healing. There was no statistically significant difference regarding index A, index C, and tissue texture after 4 and 8 weeks, which could be due to a small sample selection in each group and insufficient dispersion within each group (Table 1).

**Table 1:** Histomorphometrical analysis of regenerated bone in horizontal (Index A) and vertical (Index C) dimensions in the tibia rabbits after 4 and 8 weeks implantation. There was no significant difference between groups (n = 32).

Group	Index	Time point	Average	Median	Standard deviation	Significant difference
**TiZr**	A	4	3.8	4	0.33	NO
**TiZr**	A	8	3.5	3	0.83	NO
**TiZr**	C	4	5.2	4	0.33	NO
**TiZr**	C	8	2.6	3	0.51	NO
**TiZr-Doxy**	A	4	3.25	3	0.5	NO
**TiZr-Doxy**	A	8	3.87	2	0.35	NO
**TiZr-Doxy**	C	4	2.25	4	0.5	NO
**TiZr-Doxy**	C	8	2.62	3	0.51	NO

### 3.4 Histological and histomorphometrical analyses of dog study

**Fig. 8:**
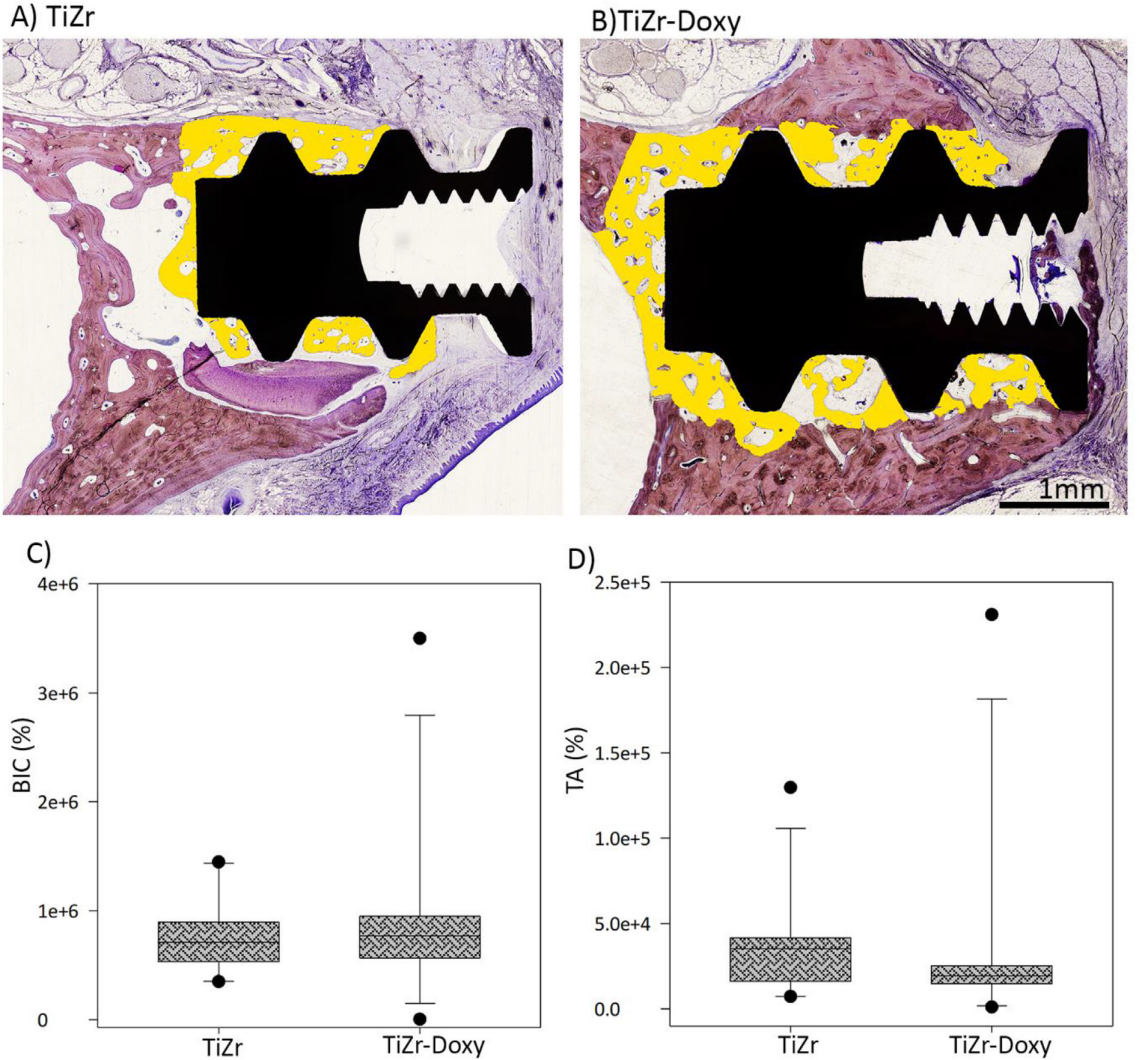
The representative images of histology and histomorphometry analyses in the dog study. **A&B)** Levai Laczko stained tissues for both doxycycline coated and uncoated groups in 4x magnification. The yellow selected region is the new bone formation in contact with bone chamber TiZr implants. **C&D)** Histomorphometrical analysis of BIC and TA percentage 4 weeks after implantation.

Regarding implant osseointegration, the Levai Laczko stained dental tissues indicated no significant difference between coated and uncoated groups 4 weeks after bone chamber implantation in dogs (Fig. 8A&B). In addition, Fig. 8C&D demonstrates One-Sample Signed Rank Test results for BIC and TA percentage 4 weeks after implantation. Normality test (Shapiro- Wilk) failed for both parameters using P < 0.05. When the normality test failed, we used a type of (ANOVA)-test called “Kruskal-Wallis One Way Analysis of Variance on Ranks”. There was no statistically significant difference between two groups.

## 4 Discussion

The current progress in producing implants has given a higher than 90% success rate of dental implantation surgery within five years [29]. Despite these improvements, there are still high-risk patient groups, where one could observe lower success rates due to several reasons such as smoking habits, radiation therapy or systematic disorders and weakened immune systems [30]. An increasing number of elderly patients also prefer dental implants and implant-retained partial dentures over conventional dentures, which create more complex patient groups [31]. Although having a good oral hygiene is a prerequisite before any surgery can take place, some patients push to the side oral hygiene when it comes to the self-care and neglect its importance after implantation, which could cause infection after implantation. The need for improving dental implants in these high-risk patient groups is caused by many factors, including general poor bone density, wound healing disruptions or high risk for infections. For this reason, substantial scientific effort has been directed toward treatments that can increase the success rate of implants in poor quality bone. In the case of diabetes a review study concluded that there is a clear benefit using preoperative antibiotics in both type II diabetics and non-diabetics [32]. For instance, Morris et al. [33] found that in the non-type II diabetic group, the success rate of implants placed with preoperative antibiotics is 4.5% higher than implants without antibiotics. This suggests that systemic application of antibiotics could improve the success rates of dental implants for these patients [33]. The factors that have to be controlled in this context are the stimulation of bone growth around the dental implant for improving osseointegration and decreasing infection risk at the implant site [34]. Coating antibiotics on the implant surface could not only prevent the biofilm formation and systematic adverse effects of drugs, but also it might improve the implant osseointegration abilities [35,36].

Hence, we developed a local drug delivery system through coating doxycycline on the TiZr implants for controlling its release in a controlled manner [34]. Results from both animal studies supported our hypothesis that adding a layer of doxycycline coating layer would not worsen the foreign body responses to titanium-based implants in healthy bone conditions. The results demonstrated that the designed system was able to sustain a sufficient drug concentration to or above the essential dosage for stimulating anti-collagenase activity and osseointegration. Compared to the neutral pH, higher dosage of doxycycline released at pH 3, which might be because of quicker dissolution of the coating layer in an acidic environment [37]. Because the objective of this study was to have doxycycline available in the presence of infection, we immersed the implants in the acidic pH solution and the obtained results were acceptable. We did not observe any significant burst release in the first 24h, which could be due to its strong chemical binding to the implant surface. The FE-SEM and profilometer measurements exhibited no significant difference in the SLActive® surface topography or morphology after doxycycline coating. Hence, one could conclude that after coating doxycycline the favorable SLActive® surface for dental applications preserved its physical properties. However, because of the complex physiological conditions at body (more enzymes, fluid flow, different pH); we could not compare the results of this simplified *in vitro* release model with in vivo findings. One could expect different release curves for doxycycline when placed in bone than what we measured on the lab bench. In our previous study, we evaluated the doxycycline integration depth by two-dimensional micro-CT in vivo [10]. The results showed a high depth integration for doxycycline indicating the drug release and its transport into the defect region [10].

The results obtained from SEM and confocal microscopes confirmed the physical presence of doxycycline layer on the surface; however, the surface morphology did not significantly change after coating. Because some studies have reported that not only Sa, but also Ssk, Sku and Sci are important factors in determining the implant osseointengation [38,39], we studied all of their changes after coating to ensure that surface morphology did not change the bone-tissue interplay.

The used animal models and implants shapes in this study are recognized as validated models for evaluating dental implants [[40], [41], [42]]. R ø nold et al. [24,43] used the rabbit model to develop the Dentsply-Sirona Osseospeed® implant [24,43]. In the current rabbit model, a modification to this model was made by making a large hole into the lumen of the rabbit tibia. This was in order to evaluate the new bone formation adjacent to a coated titanium surface. On the other hand, we used dogs for the second animal study as they are among the most well-established animal models for dental research such as studying dental naturally occurring gingivitis and periodontitis [44,45]. Periodontal tissues and the size of teeth in dogs are largely comparable to those in humans [[24], [43]].

Regarding the shape of implants, dental implants are generally cylindrical or threaded shaped screws. These threads are designed to provide primary stability for the implant before attaching a prosthesis. They also increase the influence of old bone bonding through interlocking bone with the implant surface. Consequently, when researchers evaluate the new bone attachment and ingrowth to implants in vivo, they might face incorrect positive results. In this study, we used coin shaped and bone chamber implants, which because of their shape, the retention between the implant and bone is only reliant on the reactions between newly formed bone and implants. Therefore, the following histological and histomorphometrical evaluations of the newly bone formation around the implant surface are more reliable [[24], [43]]. One might question the primary stability of these non-threaded implants used in this study. In the rabbit study, we used a metal band for fixing coins in the defect site and providing the primary stability. In addition, the primary stability of bone chamber implants are provided by the press-fit of the implants with the bone walls of the prepared implant beds [46]. We should address that in this study, we did not use any threaded shaped screws and the diameter of burr was equal to the diameter of implants (Ø = 4.2 mm). However, in the threaded shaped screws the diameter of burr is less than the diameter of screws. One limitation of this study could be that we did not include threaded shaped screws to compare with bone chamber implants.

Regarding the TiZr implant biocompatibility and osseointegration, the histological and histomorphometrical analyses of the both animal studies exhibited no significant difference between doxycycline coated and uncoated groups. Although coating doxycycline might not have significant positive effects on the implant osseointegration, it could be a promising solution for controlling host response locally in high-risk patient groups. In this study, animal animals with no systematic disorders (such as diabetic, osteoporosis and weakened immune system) was used, which may reduce the ability to identify possible significant differences after coating doxycycline. Consequently, future studies can focus on using animals with systematic disorders to evaluate the possible effects of doxycycline coating layer on minimizing infection locally after implantation. In addition, the sample size of this study might be a reason for not observing any statistically significant difference after coating, which was owing to the ethical issues related to using animals that limited the number of animals we used in this study to 6 for dogs and 8 for rabbits.

## 5 Conclusions

To sum up, *in vitro* tests confirmed the physical presence of doxycycline and its controlled release on the TiZr implant surface. The both animal studies indicated no significant differences between doxycycline coated and uncoated groups in both animal studies after 4 and 8 weeks healing periods in rabbits and 4 weeks for dogs. Therefore, we successfully designed a system for coating doxycycline on the TiZr implant surface without reducing its biocompatibility and osseointegration abilities. We concluded that there was no negative effect on osseointegration of dental implants by coating the surface with doxycycline. However, further research on evaluating its possible release effects on controlling infection in animal models with systematic disorders could provide more information on its efficacy for using as a coating layer on titanium-based implants to control post-operative infections in high-risk patient groups.

## CRediT authorship contribution statement

**Maryam Rahmati:** Writing - original draft, Conceptualization, Methodology, Software, Formal analysis. **Ståle Petter Lyngstadaas:** Writing - review & editing, Conceptualization, Data curation. **Janne E. Reseland:** Writing - review & editing, Resources. **Ingrid Andersbakken:** Investigation, Software. **Heidi Straume Haugland:** Investigation, Formal analysis. **Mónica López-Peña:** Writing - review & editing. **Antonio Gonzalez Cantalapiedra:** Visualization. **Fernando Maria Guzon Muñoz:** Writing - review & editing. **Håvard Jostein Haugen:** Supervision, Project administration, Funding acquisition.

## Declaration of competing interest

Håvard Jostein Haugen is one of the inventors of the patent «Dental implants for high risk patients» PCT/EP2014/060929 WO2014191399 A1 assigned to Institute Straumann Holding AG. Haugen does not receive any numeration for this patent.
